# Osteoblast Hypoxia-Inducible Factor-1α Pathway Activation Restrains Osteoclastogenesis *via* the Interleukin-33-MicroRNA-34a-Notch1 Pathway

**DOI:** 10.3389/fimmu.2017.01312

**Published:** 2017-10-16

**Authors:** Hui Kang, Kai Yang, Lianbo Xiao, Lei Guo, Changjun Guo, Yufei Yan, Jin Qi, Fei Wang, Bernhard Ryffel, Changwei Li, Lianfu Deng

**Affiliations:** ^1^Shanghai Key Laboratory for Prevention and Treatment of Bone and Joint Diseases with Integrated Chinese-Western Medicine, Shanghai Institute of Traumatology and Orthopedics, Ruijin Hospital, Shanghai Jiaotong University School of Medicine, Shanghai, China; ^2^Department of Orthopedics, Shanghai Tenth People’s Hospital, Tongji University School of Medicine, Shanghai, China; ^3^Guanghua Integrative Medicine Hospital and Institute of Arthritis Research, Shanghai Academy of Chinese Medical Sciences, Shanghai, China; ^4^Experimental and Molecular Immunology and Neurogenetics (INEM), UMR 7355 CNRS and University of Orleans, Orleans, France

**Keywords:** hypoxia-inducible factor-1α, interleukin-33, osteoblast, osteoclastogenesis, microRNA-34a-5p, Notch1

## Abstract

Functional cross-talk between osteoblasts and osteoclasts is a key process for bone homeostasis. Although osteoblast hypoxia-inducible factor-1α (HIF-1α) pathway activation results in impaired osteoclastogenesis *via* the direct regulation of osteoprotegerin (OPG), it is unclear whether there are other efficient mediators are involved in osteoblast HIF-1α pathway activation-restrained osteoclast formation. In addition to upregulated OPG, we observed that osteoblast HIF-1α activation led to increased interleukin-33 (IL-33) expression, which was found to inhibit osteoclastogenesis. Mechanistically, HIF-1α facilitates IL-33 expression by binding to −1,504/−1,500 bp on the *Il-33* promoter. IL-33, thereby, acts on bone marrow-derived monocytes (BMMs) to reduce their osteoclastic differentiation. Moreover, microRNA-34a-5p (miR-34a-5p)-inhibited Notch1 activation was observed to play a central role in this process. Thereby, the identification of IL-33-miR-34a-5p-Notch1 pathway in the inhibitory effect of osteoblast HIF-1α pathway on osteoclastogenesis uncovers a new mechanism for understanding the effects of HIF-1α on bone remodeling.

## Introduction

As a dynamic tissue, bone undergoes life-long remodeling: osteoblasts regulated bone formation and osteoclasts mediated bone destruction or resorption (1). The functional cross-talk between osteoblasts and osteoclasts is a key process for bone homeostasis. Both defects in the cells and obstruction of intercellular communication can lead to debilitating bone disorders such as osteopetrosis, osteoporosis, rheumatoid arthritis, and even tumor metastasizing to the bone (2, 3).

It has been well established that osteoblasts play a crucial role in the process of differentiation and maturation of osteoclasts by producing several factors that bind directly to osteoclastic progenitors (2). Besides expressing macrophage colony-stimulating factor (M-CSF) and receptor activator of nuclear factor kappa-B ligand (RANKL) to promote the survival, differentiation, and activation of osteoclast progenitors, osteoblasts also secrete osteoprotegerin (OPG), which acts as a decoy receptor, highly specific to RANKL, that inhibits the differentiation of osteoclasts (4). When under the effects of certain physical and chemical factors, osteoblasts can also express some cytokines to regulate osteoclast formation (5, 6). Studies have also revealed that differentiated osteoblasts can express interleukin-33 (IL-33) to block osteoclast formation from bone marrow-derived macrophages (7, 8), and that rates of osteoclastogenesis are higher in IL-33 receptor-ST2 deficient mice (8). However, whether IL-33 is involved in the cross-talk between osteoblasts hypoxia-inducible factor (HIF)-1α pathway activation and osteoclasts formation is unknown.

During bone development, osteoblasts are exposed to mild hypoxic conditions. Studies have shown that the oxygen-sensing pathway related proteins, such as von Hippel–Lindau tumor suppressor protein (VHL), prolyl hydroxylase-domain proteins (PHDs), and HIFs, are all expressed in osteoblasts (9). Our results reveal that HIF-1α in osteoblasts accelerates angiogenesis as well as osteogenesis *via* increasing vascular endothelial growth factor (VEGF) expression during bone development (10, 11), and upregulated VEGF expression in *Vhl*-deficient osteoblasts facilitates bone marrow stromal cells (BMSCs) proliferation and osteogenic differentiation (11). In addition to coordinating osteoblastic–angiogenic coupling, both VHL/HIF and PHD/HIF signaling pathways in osteoblasts have been proved to facilitate bone homeostasis by regulating osteoclastogenesis through the direct regulation of OPG (12, 13). Thus, besides increasing angiogenesis or osteoblastic activity, hypoxia/HIF-1α pathway activation pormotes bone development may also *via* disturbing osteoblast and osteoclast coupling and thereby dampens osteoclast activation. However, whether there are other efficient mediators besides OPG are involved in osteoblast HIF-1α pathway activation-restrained osteoclastogenesis still need for further exploration.

In the present study, we proved that, in addition to OPG, IL-33 is essential for osteoblast HIF-1α pathway-inhibited osteoclastogenesis. Furthermore, we provided evidence that IL-33 reduces osteoclastogenesis *via* the miR-34a-5p-Notch1 pathway.

## Materials and Methods

### Reagents

Alpha modification of Eagle medium (α-MEM), penicillin/streptomycin and fetal bovine serum were purchased from Gibco-BRL (Sydney, NSW, Australia). Recombinant soluble mouse M-CSF (Catalog#315-02) and mouse RANKL (Catalog#315-11) were purchased from Peprotech (USA). Primary antibodies of anti-HIF-1a (NB100-105), anti-Notch1 (NB100-78486), anti-Hes-1 (NBP1-30912), and anti-β-actin were purchased from Novus Biologicals (USA). IL-33 (3626-ML), anti-IL-33 (MAB3626), IL-33-neutralizing antibody (AF3626), OPG-neutralizing antibody (AF459), recombinant jagged-1 protein was provided by R&D Systems (USA). Desferrioxamine (DFO), the tartrate-resistant acid phosphatase (TRAP) staining kit was bought from Sigma Aldrich (St Louis, MO, USA).

### Generation of Conditional Knockout Mice

Generation and genotyping analysis of the Vhl^flox/flox^ and OC-Cre transgenic mice have previously been described (10). Briefly, Vhl^flox/flox^ transgenic mice and mice containing osteocalcin (OC) promoter-driven Cre recombinase were intercrossed to generate osteoblast *Vhl* conditional knockout (*Vhl*-CKO) mice. Therefore, for all experiments with these mice, age- and sex-matched Cre-recombinase positive mice were used as VHL-CKO group while the Cre-recombinase negative mice were used as VHL-CON group. All procedures involving mice were performed in accordance with the Shanghai Jiaotong University Animal Study Committee.

### Primary Osteoblasts Isolation and Conditioned Medium (CM) Preparation

The preparation of CM was generated as previously described (11). The calvariae of newborn Vhl^flox/flox^ transgenic mice was used for primary osteoblasts isolation by serial round of digestion with 1.8 mg/ml type I collagenase (Sigma). To disrupt *Vhl in vitro*, the osteoblasts after three passages were infected with control adenovirus (Ad-GFP) or adenovirus expressing Cre recombinase (Ad-CRE, Vector Biolabs) at an MOI of 100. After incubation for 48 h, real-time RT-hypoxia response element (PCR) and western blot were conducted to detect the knock down efficiency of *Vhl* in osteoblasts.

Before the collection of culture medium of osteoblasts infected with Ad-GFP (CM-GFP) or culture medium of osteoblasts infected with Ad-CRE (CM-CRE), the cells were changed to incubate with α-MEM without serum or penicillin/streptomycin. In this study, the CM-GFP and CM-CRE were harvested after 24, 72, and 120 h and centrifuged at 3,000 rpm for 15 min, respectively, and then stored at −80°C. Additionally, 50% CM-GFP or CM-CRE was used in the following experiment and was not given special labeling in the figures.

### *In Vitro* Osteoclastogenesis and Osteoclast Activity Assay

Tibiae and femurs of 8-week-old mice were used for primary bone marrow-derived monocytes (BMMs) isolation by flushing the bone marrow with α-MEM. BMMs were cultured in α-MEM for 16 h, then non-adherent cells were harvested and cultured in CM-GFP or CM-CRE complete medium supplemented with 50 ng/ml M-CSF for 3 days. Adherent cells were harvested as osteoclast progenitors and were further cultured with CM-GFP or CM-CRE containing M-CSF (30 ng/ml) and RANKL (50 ng/ml) for another 3–5 days. To investigate the other mediators besides of OPG in the inhibitory effect of CM-CRE on osteoclastogenesis, cells were differentiated withthe OPG-neutralizing antibody (200 ng/ml) as previously described (14). For the research of IL-33 on osteoclastogenesis, various concentrations of IL-33 or IL-33-neutralizing antibody were used to treated the BMMs. TRAP staining was performed according to the manufacturer’s instructions (Sigma-Aldrich). TRAP-positive cells with three or more nuclei were counted under a microscope (15). For the *in vitro* TRAP activity assay, cells were lysed by passive lysis buffer (Promega), 40 µl of supernatant was transferred to a 96 well plate and the test of TRAP activity was made with use of a Tartrate Resistant Acid Phosphatase Assay Kit (Beyotime Biotechnology, China). Absorbance at 405 nm was measured in a Tecan plate reader after 5~10 min of incubation. BCA Protein Assay Kit (Beyotime Biotechnology, China) was used to measure the protein concentration in each well. TRAP activity in each well was normalized to the corresponding protein concentration.

### Bone Resorption Assay and F-Actin Ring Formation Assay

The bone resorption assay was carried out as previously report (16). Briefly, osteoclast progenitors were plated on bovine bone slices with a density of 8 × 10^4^ cells/mL, cultured in CM-GFP or CM-CRE supplemented with 30 ng/mL M-CSF and 50 ng/mL RANKL. Osteoclasts adherented on the slices were removed out by sonication after 7 days of culture; the resorption pits stained with toluidine blue were photographed under a high-quality microscope. Resorption analysis was quantitated with the Image J software (Bethesda, MD, USA), three fields of view were randomly selected on each section.

To perform F-actin ring formation assay, osteoclasts treated with CM-GFP or CM-CRE were stained with rhodamine-conjugated phalloidin (Life Technologies) for 30 min at room temperature and then stained with 4,6-diamidino-2-phenylindole (Sigma) for nuclei detection. The F-actin ring formation was photograhped with a fluorescence microscope (Carl Zeiss, Jena, Germany), and the average number of normal F-actin rings was calculated as described (17).

### Quantitative Real-time RT-PCR

Trizol reagent (Invitrogen, Carlsbad, CA, USA) was used for total RNA extraction. For reverse transcription of mRNA and miR-34a-5p, 1 µg of total RNA was used for reverse transcription with Prime-Script RT reagent kits: Cat#RR036A and Cat#RR037A (TaKaRa Biotechnology, Japan), respectively. Quantitative real-time PCR was performed to amplify the cDNA by the SYBR Premix Ex Tag kit (TaKaRa Biotechnology, Japan) and ABI 7500 Sequencing Detection System (Applied Biosystems, Foster City, CA, USA). β-actin and U6 were used as endogenous control for quantitation of mRNAs and miR-34a-5p, respectively. The specific primer sequences for real-time RT-PCR were described in Table S1 in Supplementary Material.

### Bone Histomorphometry and Immunohistochemistry (IHC)

Bone tissues were embedded with paraffin after decalcification. 5 μm-thick sections were stained with hematoxylin and eosin (H&E) or TRAP according to standard methods, respectively. The ratio of osteoclast numbers to the trabecular bone surface (N.Oc/BS) was quantified with Osteomeasure Analysis System (Osteometrics, Atlanta, GA, USA) on TRAP-stained sections at 200× magnification.

For IHC analysis, deparaffinized sections were incubated with 3% H_2_O_2_ for 15 min, and then treated with 5% BSA for 10 min. Next, the sections were incubated with HIF-1α (1:50) and IL-33 (1:100) primary antibodies overnight at 4°C, respectively. Followed by incubated with biotin conjugated secondary antibodies, and visualized with the streptavidin-biotin staining technique. Nucleus was stained with hematoxylin and the slides were photographed by a microscope (ZEISS, AXIO).

### MicroRNA (miRNA) Transfection

Cells were transfected with synthetic miRNA for anti-miR-34a (*miR-34a* antagomir) and its negative control (NC) using Lipofectamine™ RNAiMAX (Life Technologies). The *miR-34a* antagomir and NC were designed and synthesized by GenePharma (Shanghai, China). The sequences of *miR-34a* antagomir and NC used were as follows: *miR-34a* antagomir: 5′-CCAGCTAAGACACTGCCA-3′, NC: 5′-CAGUACUUUUGUGUAGU-3′. After incubation for 48 h, the cellular lysates were harvested to analysis the expression of genes of interest.

### Western Blot Analysis

Cells were lysed with RIPA buffer supplemented with protease inhibitor cocktail (Sigma, St Louis, MO, USA). BCA protein assay kit (Beyotime Biotechnology, China) was used to determine the protein concentration. About 20 µg proteins were loaded, separated in 10% SDS-PAGE gels, and then transferred to PVDF membranes (Millipore, Bedford, MA, USA). Subsequently blocked with 5% skimmed milk solution and labeled with primary antibodies at 4°C overnight. Followed by immunoblot with peroxidase-conjugated secondary antibodies, the bands were visualized by the enhanced chemiluminescence detection system. β-actin was used as the endogenous control. Signal intensities were quantified using Image J software.

### Enzyme-Linked Immunosorbent Assay (ELISA)

The concentration of IL-33 in osteoblasts culture supernatant were detected using Mouse IL-33 Quantikine ELISA Kit (M3300, R&D Systems) according to the manufacturer’s instructions.

### Flow Cytometry Analysis for Osteoclast Progenitors

After incubation for the indicated times, osteoclast progenitors were harvested and made to a single-cell suspension and followed by incubated with FITC anti-CD11c (eBioscience), APC anti-CD206 (eBioscience) antibodies. Flow cytometric analysis was performed using FACS LSR II (BD Biosciences, San Jose, CA, USA).

### Luciferase Reporter Assay

The −2,172~−134 bp of IL-33 encoding region was selected as the promoter. The promoter region was analyzed in the JASPAR core database, which revealed the presence of putative hypoxia response element (HRE) at −1,504/−1,500 bp relative to the transcription start site on IL-33 promoter, whose specific sequences is ACGTG. Then, IL-33 promoter reporter (−2,172~−134 bp, including the WT and mutants with putative binding site detection) was directly cloned into a pGL3-Basic luciferase vector. Primers used for amplifying mouse IL-33 WT and mutated promoters by PCR were as follows: WT, forward: 5-CTACTCACTAGCGCATGATTCAG-3, reverse: 5-CGATGATTCTGCCGTGATTTCTCC-3; mutant with putative binding site deletion, bp −1,504 to bp −1,500, forward: 5-TAACTTACTCTTCCTACGGGAGGGTAGTCACTCT-3, reverse: 5-AGAGTGACTACCCTCCCGTAGGAAGAGTAAGTTA-3. After 24 h of Ad-CRE incubation, mouse primary osteoblasts were seeded into 24-well plates that were co-transfected with different plasmids (firefly reporter constructs containing the WT or mutant IL-33 promoter and a Renilla-expressing plasmid). Firefly and Renilla luciferase activities were measured 24-h post-transfection by a Dual Luciferase Assay System (Promega).

### Statistical Analysis

Data were collected from three or more independent experiments and expressed as mean ± SEM. A two-sided Student’s *t*-test was used to analyze the difference between groups. One-way analysis of variance was performed to show the difference between groups. *P* < 0.05 was considered significantly different.

## Results

### Osteoblast HIF-1α Pathway Mediates Osteoclastogenesis Partly through IL-33

**Figure 1:**
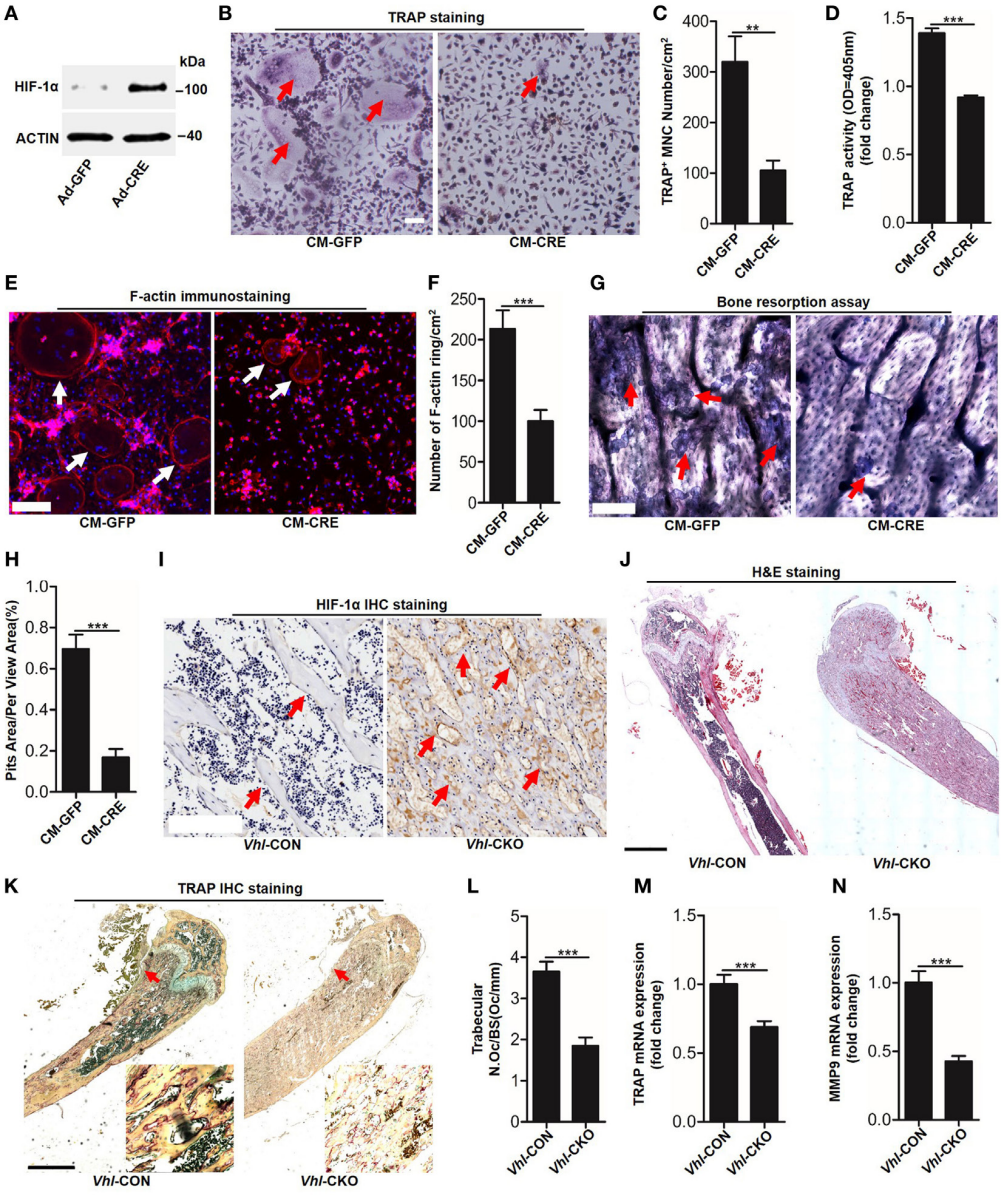
Hypoxia-inducible factor (HIF)-1α activation in osteoblasts restrains osteoclastogenesis. **(A)** Western blot analysis of HIF-1α in osteoblasts after transfected with Ad-GFP or Ad-CRE. **(B–D)** Bone marrow-derived monocytes (BMMs) osteoclastic formation and tartrate-resistant acid phosphatase (TRAP) activity assay after treating with the CM-CRE or CM-GFP. Red arrows indicated multinucleated osteoclasts. **(E–H)** F-actin staining and resorption pit formation in BMMs after treating with the CM-CRE or CM-GFP. White arrows indicated F-actin rings, red arrows indicated resorption pits. **(I)** Immunohistochemical staining of HIF-1α in trabecular bone sections from *Vhl*-CON and *Vhl* conditional knockout (*Vhl*-CKO) mice. **(J)** H&E staining of trabecular bone sections from *Vhl*-CON and *Vhl*-CKO mice. **(K,L)** TRAP staining of trabecular bone sections from *Vhl*-CON and *Vhl*-CKO mice. Arrows designate the region of 200× magnification shown in the lower right corner. **(M,N)** Quantification of TRAP and MMP9 mRNA levels in trabecular bone tissues from *Vhl*-CON and *Vhl*-CKO mice. Scale bars represent 100 µm. Ad-CRE represents adenovirus expressing Cre recombinase, while Ad-GFP represents the control adenovirus. CM-CRE represents the culture medium of osteoblasts transfected with Ad-CRE, while CM-GFP represents the culture medium of osteoblasts transfected with Ad-GFP. *Vhl*-CKO means Cre-recombinase positive mice while *Vhl*-CON means Cre-recombinase negative mice. **P < 0.01, ***P < 0.001. *P* values were analyzed by *t*-test.

To detect functional cross-talk between osteoblasts HIF-1α pathway activation and osteoclastogenesis, we activated the HIF-1α pathway *via* the *Cre-Loxp* system to knockdown *Vhl* in osteoblasts. Compared with the control adenovirus (Ad-GFP) treated group, transfected with the Ad-CRE in osteoblasts significantly increased HIF-1α expression (Figure 1A). Next, we examined the effect of osteoblast HIF-1α activation on osteoclast formation. TRAP staining and TRAP activity assays results revealed that compared with group stimulated with the conditioned culture medium of osteoblasts infected with Ad-GFP (CM-GFP), the conditioned culture medium of osteoblasts infected with Ad-CRE (CM-CRE) treatment strongly reduced osteoclastogenesis (Figures 1B–D). Furthermore, the F-actin ring (Figures 1E,F) and resorption pit formation (Figures 1G,H) assay demonstrated that HIF-1α activation in osteoblasts significantly inhibited mature osteoclast formation and osteoclastic bone resorption.

Consistent with the *in vitro* results, the inhibitory effect of HIF-1α activation on osteoclast formation was further demonstrated *in vivo*. Immunohistochemical staining showed that there were higher numbers of HIF-1α positive cells surrounding the trabecular bone of *Vhl*-conditional knockout mice (Cre-recombinase positive mice, *Vhl*-CKO) than the control mice (Cre-recombinase negative mice, *Vhl*-CON) (Figure 1I). Hematoxylin and eosin (H&E) staining revealed more trabecular bone formation in the *Vhl*-CKO mice than in the *Vhl*-CON mice (Figure 1J). In line with these results, TRAP staining analysis, quantification of TRAP, and MMP9 mRNA levels in trabecular bone tissues revealed that osteoclastogenesis was reduced in the *Vhl*-CKO mice compared to the *Vhl*-CON mice (Figures 1K–N).

**Figure 2:**
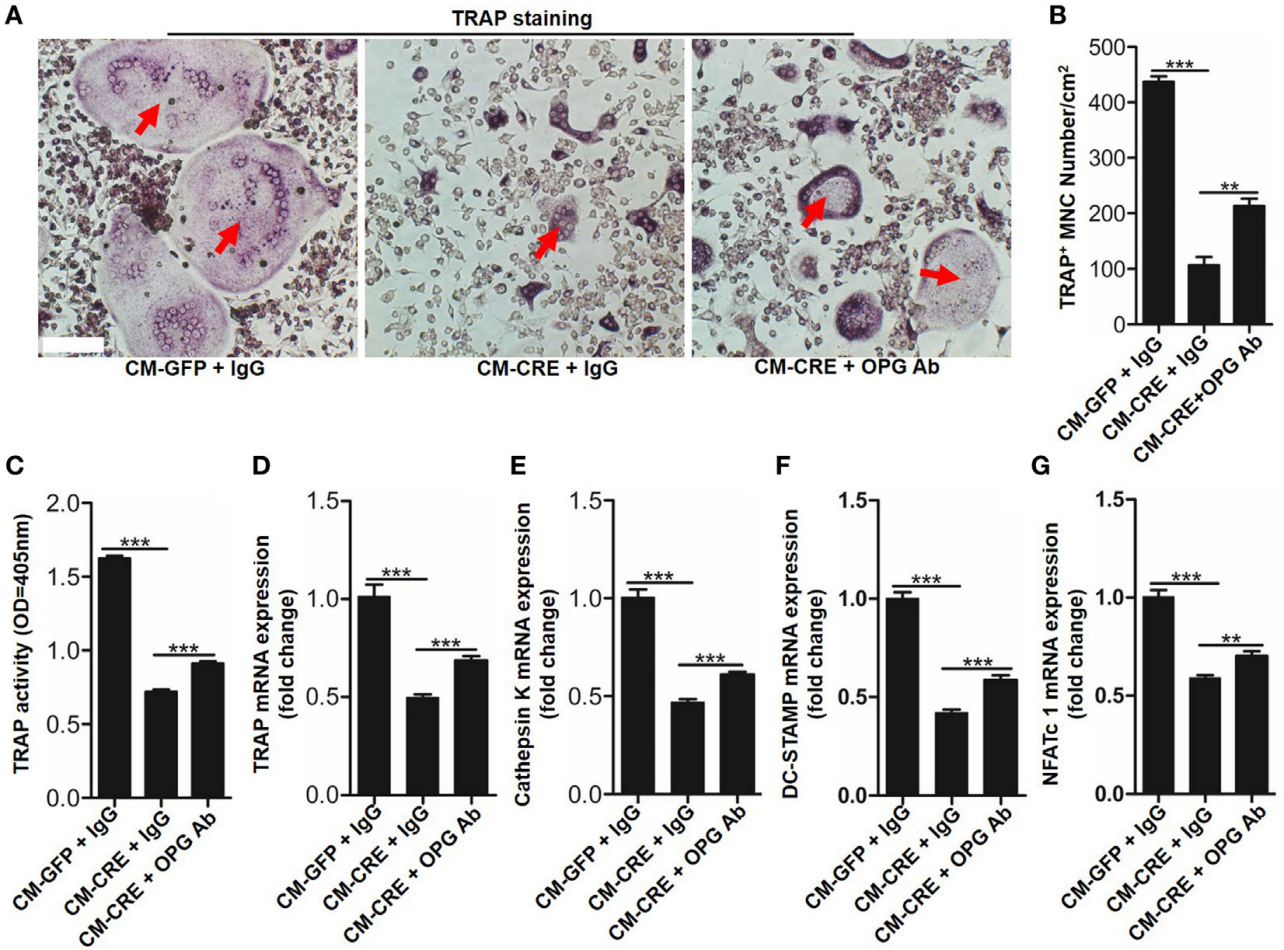
Hypoxia-inducible factor (HIF)-1α activation in osteoblasts restrains osteoclastogenesis in part through osteoprotegerin (OPG). **(A–C)** BMMs osteoclastic formation and tartrate-resistant acid phosphatase (TRAP) activity assay after treated with CM-GFP and CM-CRE in the presence of OPG neutralizing antibody. Red arrows indicated multinucleated osteoclasts. **(D–G)** Quantification of *Trap, Cathepsin K, Dc-stamp*, and *Nfatc1* expression in BMMs treated with CM-GFP and CM-CRE in the presence of OPG neutralizing antibody. Scale bars represent 100 µm. CM-CRE represents the culture medium of osteoblasts transfected with Ad-CRE, while CM-GFP represents the culture medium of osteoblasts transfected with Ad-GFP. ***P < 0.001. *P* values were analyzed by one-way ANOVA.

Osteoblasts HIF-1α signaling pathway activation by *Vhl* or PHD silencing plays a critical role in bone homeostasis by regulating osteoclastogenesis through OPG (12, 13). However, it is unclear whether osteoblast HIF-1α activation can mediate osteoclastogenesis through other “paracrine” approaches. To test it, we blocked the function of OPG by applying an OPG-specific neutralizing antibody. The results of TRAP staining (Figures 2A,B), TRAP activity assays (Figure 2C) and osteoclastogenesis related genes expression, such as *Trap* (Figure 2D), *cathepsin-K* (Figure 2E), dendritic cell specific transmembrane protein (*Dc-stamp*) (Figure 2F), and nuclear factor of activated T cells c1 (*NFATc1*) (Figure 2G) showed that OPG neutralizing antibody significantly dampened the inhibitory effect of CM-CRE on osteoclastogenesis. Simultaneously, the results also revealed that the OPG neutralizing antibody could not completely restore the osteoclast formation to the same level as the control group, which suggested that osteoblast HIF-1α pathway activation-restrained osteoclastogenesis might through other mediators.

**Figure 3:**
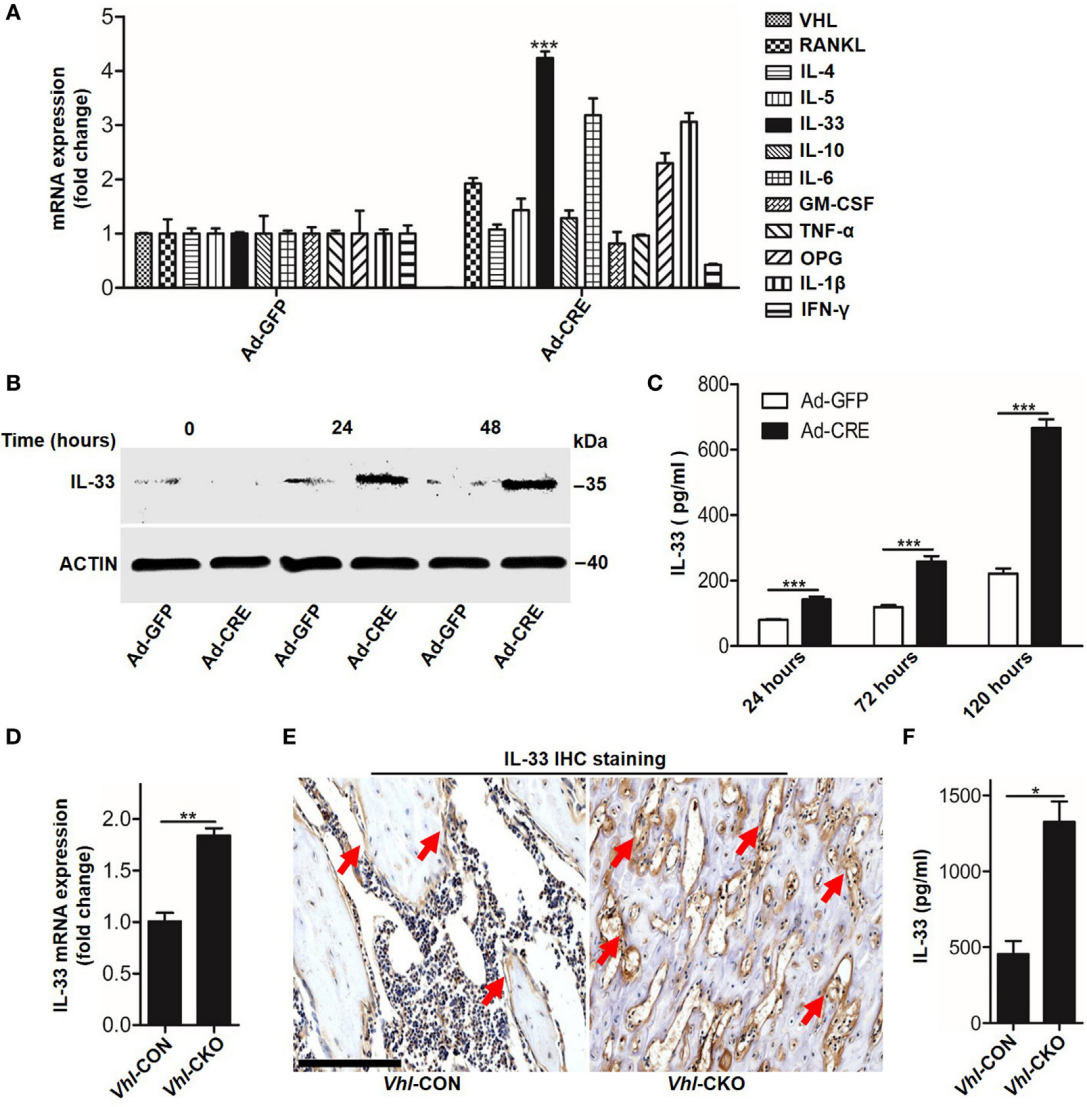
Hypoxia-inducible factor (HIF)-1α activation in osteoblasts increases interleukin-33 (IL-33) expression. **(A)** Quantification of expression of different genes in osteoblasts transfected with Ad-GFP or Ad-CRE. **(B)** Western blot analysis of IL-33 in osteoblasts at different times following Ad-GFP and Ad-CRE transfection. **(C)** Enzyme-linked immunosorbent assay (ELISA) quantification of IL-33 in the supernatant of osteoblast culture medium at different times following Ad-GFP and Ad-CRE transfection. **(D)** Quantification of IL-33 mRNA levels in trabecular bone tissues from *Vhl*-CON and *Vhl* conditional knockout (*Vhl*-CKO) mice. **(E)** Immunohistochemical staining of IL-33 in trabecular bone sections from *Vhl*-CON and *Vhl*-CKO mice. Red arrows indicated IL-33-positive cells. **(F)** ELISA quantification of IL-33 in the trabecular bone marrow cavities of *Vhl*-CON and *Vhl*-CKO mice. Scale bars represent 100 µm. Ad-CRE represents adenovirus expressing Cre recombinase, while Ad-GFP represents the control adenovirus. CM-CRE represents the culture medium of osteoblasts transfected with Ad-CRE, while CM-GFP represents the culture medium of osteoblasts transfected with Ad-GFP. *Vhl*-CKO means Cre-recombinase positive mice, while *Vhl*-CON means Cre-recombinase negative mice. **P < 0.01, ***P < 0.001. *P* values were analyzed by *t*-test.

We next sought to identify which mediators took part in osteoblast HIF-1α pathway-inhibited osteoclastogenesis. PCR screening assay results showed that besides OPG, *Vhl* silence in osteoblasts increased *Il-33* expression (Figure 3A). Increased IL-33 expression in *Vhl*-silenced osteoblasts was also demonstrated by western blot analysis (Figure 3B). As IL-33 has been proven to be an effective inhibitor of osteoclast formation by bone marrow precursors (7, 8), we predicted that IL-33 might play a role in osteoblast HIF-1α pathway-mediated osteoclastogenesis. To test our hypothesis, we first detected IL-33 secretion after *Vhl* knockdown. ELISA results showed that after 5 days of Ad*-*CRE transfection, there was approximately 600 pg/ml IL-33 in the culture medium of the osteoblasts, and the concentration of IL-33 in the control group was approximately 200 pg/ml (Figure 3C). Real-time PCR and immunohistochemical staining revealed that IL-33 levels were strongly increased in the trabecular bone of *Vhl*-CKO mice compared to those in the *Vhl*-CON mice (Figures 3D,E). Our results in Figure 1 have demonstrated that osteoclastogenesis was reduced in *Vhl*-CKO mice, and osteoclasts are formed from monocytic progenitors of the hematopoietic lineage. We then measured IL-33 concentration in the bone marrow cavity by ELISA and found that concentration of IL-33 was much higher in the *Vhl*-CKO mice than in the *Vhl*-CON mice (Figure 3F). Taken together, these results demonstrate that HIF-1α pathway activation increased IL-33 expression in osteoblasts.

**Figure 4:**
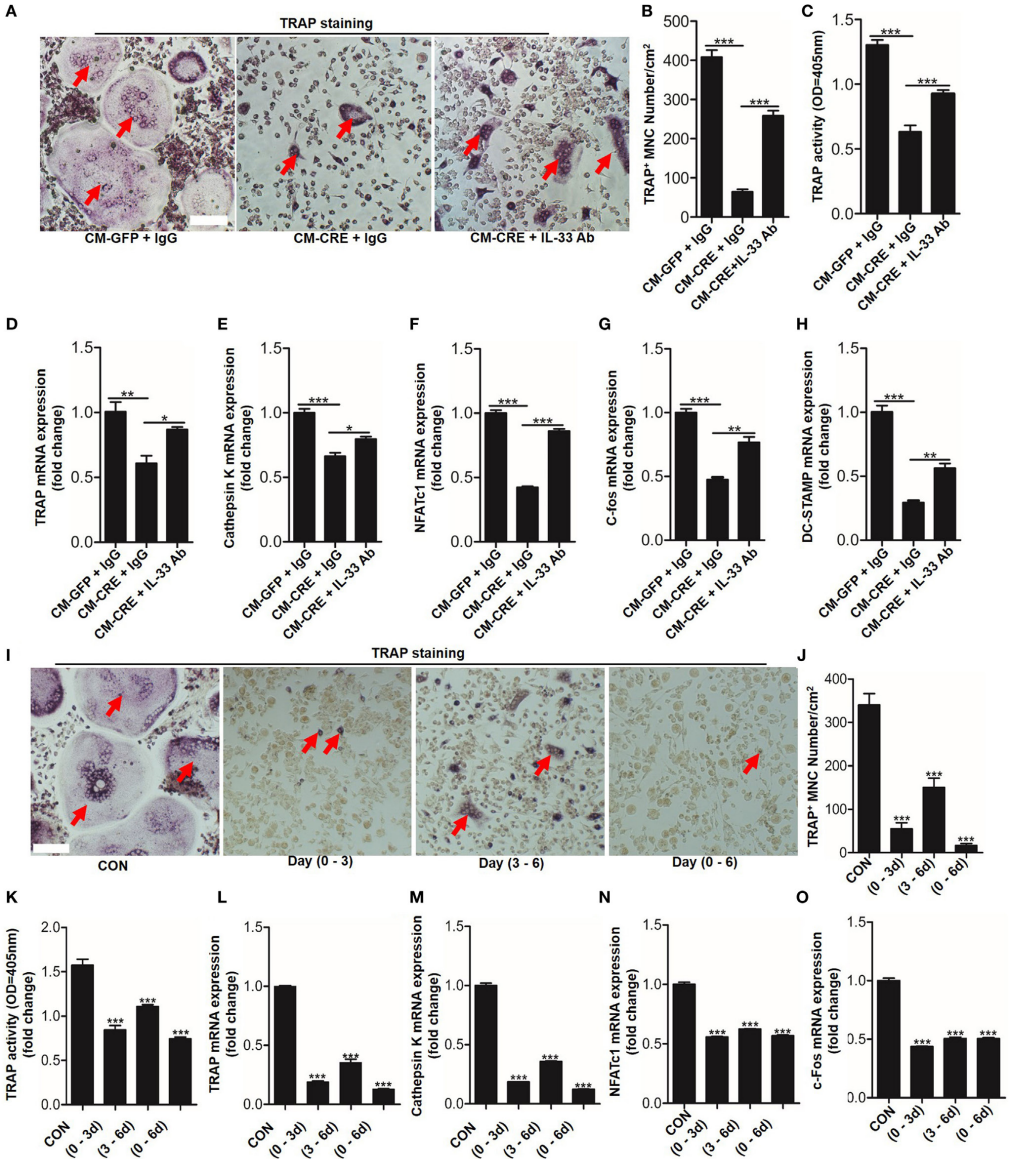
Interleukin-33 (IL-33) is involved in osteoblast hypoxia-inducible factor (HIF)-1α pathway-mediated bone marrow-derived monocytes (BMMs) osteoclastogenesis. **(A–C)** BMMs osteoclastic formation and tartrate-resistant acid phosphatase (TRAP) activity assay after treatment with the CM-GFP and CM-CRE in the presence of IL-33 neutralizing antibody. Red arrows indicated multinucleated osteoclasts. **(D–H)** Quantification of *Trap, Cathepsin K, Nfatc1, C-fos*, and *Dc-stamp* mRNA levels in BMMs treated with the CM-GFP and CM-CRE in the presence of IL-33 neutralizing antibody. **(I–K)** IL-33 (20 ng/ml) inhibits BMMs osteoclastic formation and TRAP activity at an early stage. Red arrows indicated multinucleated osteoclasts. **(L–O)** IL-33 inhibits *Trap, Cathepsin K, Nfatc1*, and *C-fos* mRNA expression in BMMs at an early stage. Scale bars represent 100 µm. CM-CRE represents the culture medium of osteoblasts transfected with Ad-CRE, while CM-GFP represents the culture medium of osteoblasts transfected with Ad-GFP. (1–3) means treated BMMs from day 1 to day 3, (1–6) means treated BMMs from day 1 to day 6, (3–6) means treated BMMs from day 3 to day 6. *P < 0.05, **P < 0.01, ***P < 0.001. *P* values were analyzed by one-way ANOVA.

Having observed that HIF-1α pathway activation increased IL-33 expression, we next sought to explore whether IL-33 was involved in osteoblast HIF-1α pathway-regulated osteoclastogenesis. We first blocked IL-33 activation in the culture medium by applying an IL-33 neutralizing antibody. TRAP staining, TRAP activity analysis, and osteoclast formation related genes expression all demonstrated that the inhibitory effect of osteoblast HIF-1α activation on osteoclastogenesis was significantly reduced when IL-33 was blocked (Figures 4A–H). Consistent with previous reports (8), we found that IL-33 inhibited osteoclast formation from BMMs in a dose-dependent manner. Concentrations as low as 5 ng/ml of IL-33 significantly reduced osteoclast formation and expression of osteoclast formation-related genes (Figure S1 in Supplementary Material). Moreover, we found that IL-33 inhibited the process of osteoclastogenesis at an early stage, as adding IL-33 in the early stage (0–3 and 0–6 days) had a stronger inhibitory effect on osteoclast formation, TRAP activity and expression of osteoclast formation-related genes than in the late stage (3–6 days) (Figures 4I–O). In addition, the increased percentage of M2 macrophages (CD206^+^) and the decreased proportion of M1 macrophages (CD11C^+^) revealed that IL-33 and CM-CRE facilitates M2 macrophages differentiation of BMMs (Figure S2 in Supplementary Material), which is also consistent with previous report (8). Taken together, these data demonstrate that IL-33 is involved in osteoblast HIF-1α pathway activation-inhibited osteoclastogenesis.

### HIF-1α Mediates IL-33 Expression by Regulating *Il-33* Promoter Activity

**Figure 5:**
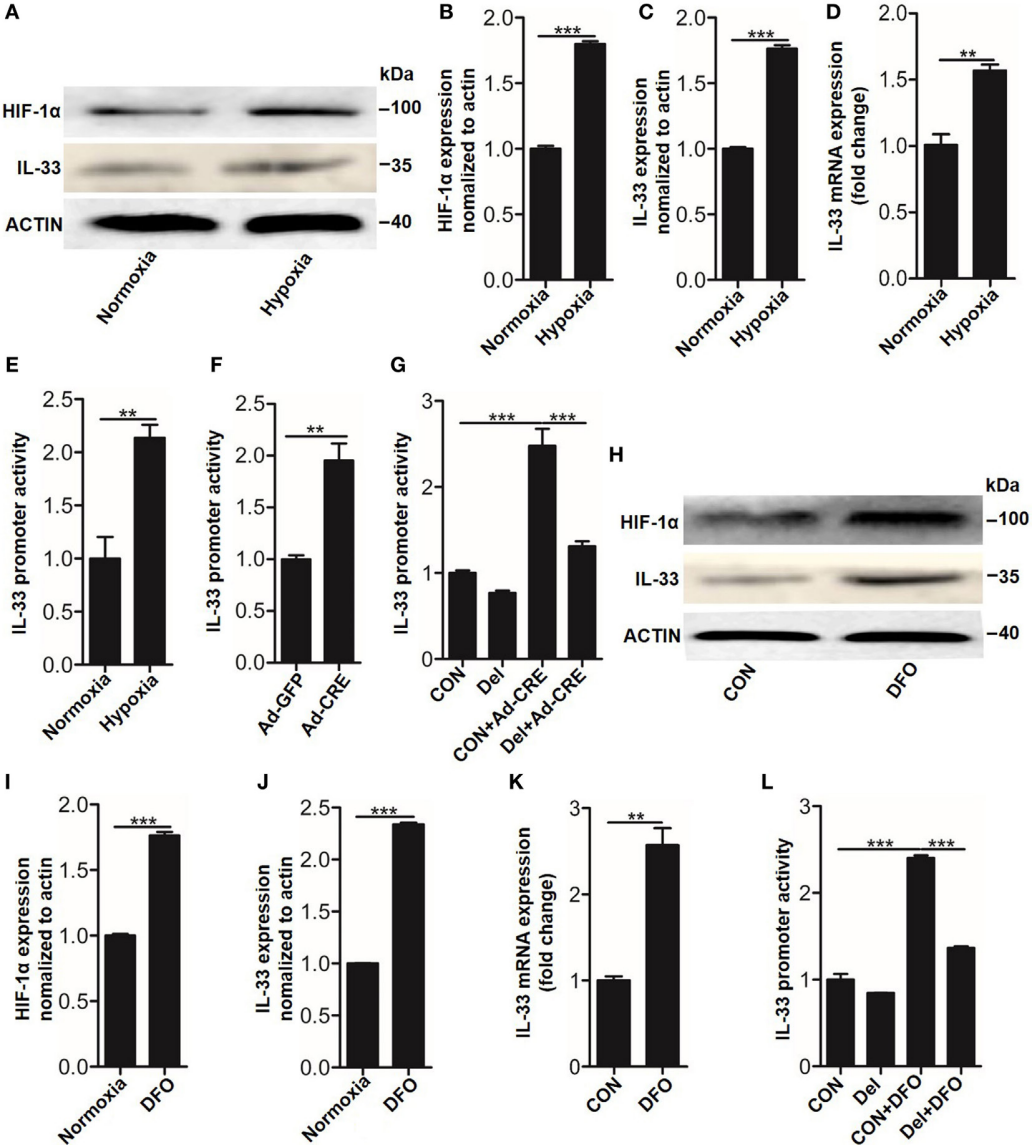
Hypoxia-inducible factor (HIF)-1α regulates interleukin-33 (IL-33) expression by binding to the promoter region of *Il-33*. **(A–D)** HIF-1α and IL-33 expression in osteoblasts under hypoxia. **(E,F)** *Il-33* promoter activity detected by the dual-luciferase reporter gene assay system in osteoblasts under hypoxia and after *Vhl* knockdown. **(G)** *Il-33* promoter activity induced by Ad-CRE and Ad-GFP in osteoblasts with or without putative HIF-1α binding site deletion in the *Il-33* promoter. **(H–K)** HIF-1α and IL-33 expression in osteoblasts induced by desferrioxamine (DFO). **(L)** *Il-33* promoter activity induced by 12 µM DFO in osteoblasts with or without putative HIF-1α binding site deletion in the *Il-33* promoter. Ad-CRE represents adenovirus expressing Cre recombinase, while Ad-GFP represents the control adenovirus. Del represents the constructs of IL-33 promoter with putative binding site detection. **P < 0.01, ***P < 0.001. *P* values were analyzed by *t*-test in **(B–D)** and one-way ANOVA in **(E,G,H)**.

Having observed that IL-33 was essential for osteoblast HIF-1α pathway-inhibited osteoclastogenesis, we next sought to explore the underlying mechanisms by which the HIF-1α pathway regulates IL-33 expression. As *Vhl* silencing lead to HIF-1α accumulation in the nucleus, where it forms a dimer with the HIF-1β subunit through its bHLH-PAS domain and binds to the promoter region of target genes to facilitate their expression, we hypothesized that HIF-1α might bind to the promoter of *Il-33* to promote its expression. To test this hypothesis, we used the dual-luciferase reporter gene assay system to detect promoter activity of IL-33 in the presence of HIF-1α activation. The results showed that in parallel with HIF-1α (Figures 5A,B), IL-33 (Figures 5A–D), and VEGFA (Figure S3 in Supplementary Material) upregulation, *Il-33* promoter activity was also significantly increased under hypoxia (Figure 5E) or Ad-CRE transfection (Figure 5F). We next sought to identify the (HRE) in the *Il-33* promoter. Analysis of mouse *Il-33* promoter sequence using the JASPAR core database (18) revealed the presence of one putative HIF-1α binding site at −1,504/−1,500 bp on the mouse *Il-33* promoter, whose specific sequence is ACGTG. To further examine if the predicted binding site is necessary for IL-33 promoter regulation by HIF-1α, we deleted the putative binding site. Cells were transfected with the WT and deleted constructs and luciferase activity was measured following Vhl-CRE adenovirus transfection in osteoblasts. The results showed that −1,504/−1,500 bp deletion caused a decrease in *Il-33* promoter activity compared to the WT promoter (Figure 5G). Since DFO has been proven to be a potent HIF-1α activator, acting by inhibiting prolyl hydroxylases in osteoblasts (19), we used DFO as an inducer of HIF-1α in osteoblasts. The results showed that HIF-1α and IL-33, as well as *Il-33* promoter activity, were abundantly increased in the osteoblasts in response to DFO stimulation (Figures 5H–K). However, the promoter activity induced by DFO was significantly reduced after the −1,504/−1,500 bp deletion (Figure 5L). Together, these results predict that HIF-1α regulates *Il-33* promoter activity by binding to −1,504/−1,500 bp on the *Il-33* promoter.

### IL-33 Mediates BMMs Osteoclastic Differentiation *via* the MicroRNA-34a-Notch1 Pathway

**Figure 6:**
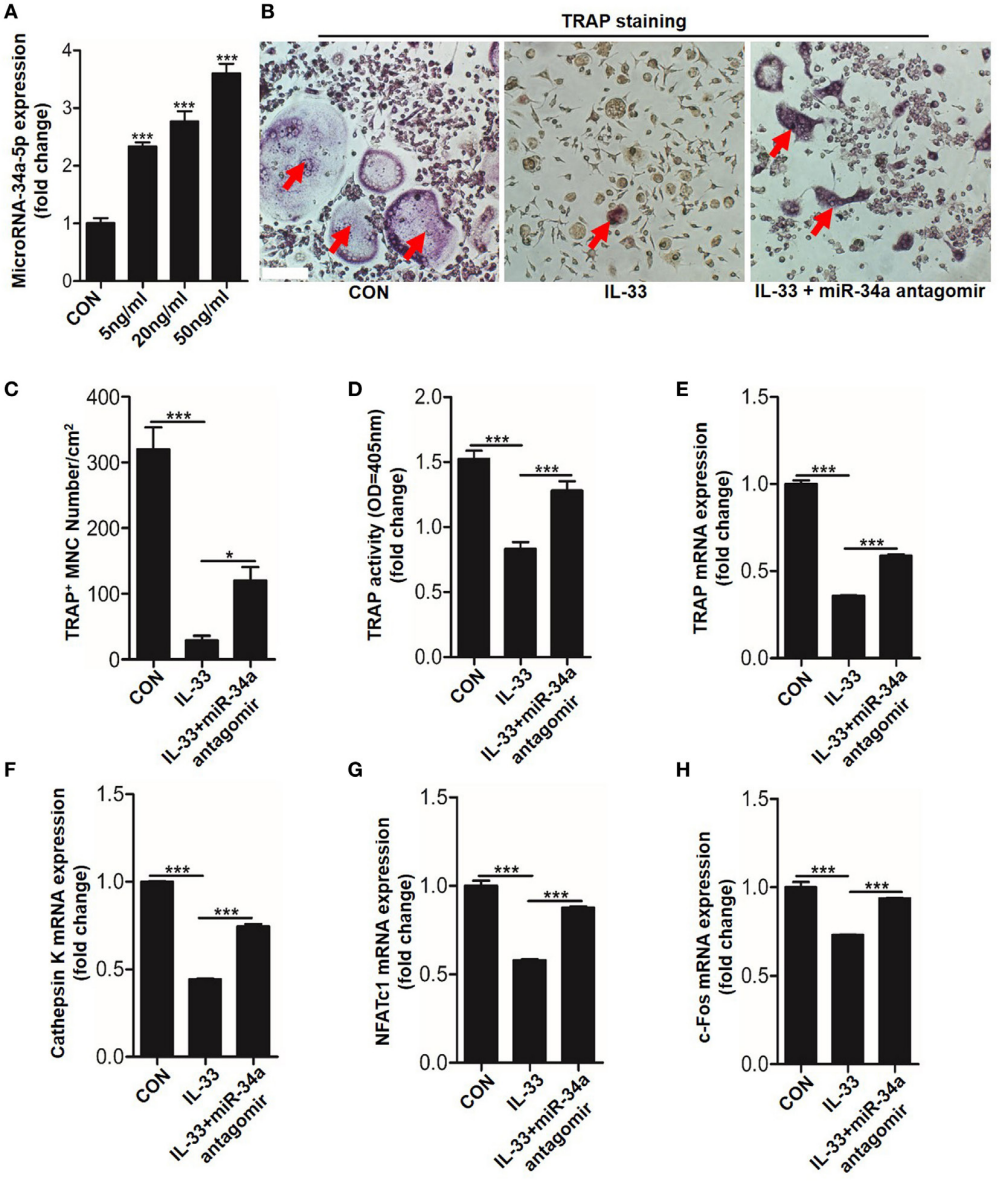
Interleukin-33 (IL-33) restrains bone marrow-derived monocytes (BMMs) osteoclastogenesis by increasing miR-34a-5p expression. **(A)** Quantification of miR-34a-5p expression induced by different doses of IL-33 in osteoclast progenitors. **(B–D)** BMMs osteoclastic formation and tartrate-resistant acid phosphatase (TRAP) activity assay after treatment with 20 ng/ml IL-33 in the presence of miR-34a-5p antagomir. Red arrows indicated multinucleated osteoclasts. Scale bars represent 100 µm. **(E–H)** Quantification of *Trap, Cathepsin K, Nfatc1*, and *C-fos* expression in osteoclast progenitors treated with 20 ng/ml IL-33 in the presence of miR-34a-5p antagomir. *P < 0.05, ***P < 0.001. *P* values were analyzed by one-way ANOVA.

Recently, studies have shown that non-coding miRNAs play an important role in osteogenesis (20–22). However, whether these molecules are implicated in the process of IL-33-repressed osteoclastic differentiation is still unknown. To explore this issue, the expression of 27 miRNAs, which are involved in BMMs differentiation, were quantitated in response to 20 ng/ml IL-33 stimulation in osteoclast progenitors. Among these miRNAs, 8 were upregulated, 18 were downregulated, while miRNA-224-5p was detected with no significant change (Figure S4 in Supplementary Material). Interestingly, we found the increase of miR-34a-5p was most pronounced among these upregulated miRNAs (Figure S4A in Supplementary Material). Given that miR-34a-5p has been proved to work as a suppressor in osteoblast differentiation (20), we hypothesized that miR-34a-5p might be involved in IL-33-restrained osteoclast formation. Quantification mRNA expression results showed that IL-33 induced miR-34a-5p expression in a dose-dependent manner in osteoclast progenitors (Figure 6A). TRAP staining and TRAP activity assay results revealed that the inhibitory effect of IL-33 on osteoclast formation was strongly reduced in the presence of miR-34a-5p antagomir (Figures 6B–D). Lastly, expression of osteoclast formation related genes, such as *Trap, capthekin-K, Nfatc1*, and *C-fos*, further demonstrated that IL-33 inhibited the process of osteoclastogenesis *via* miR-34a-5p, as these genes that decrease expression in the IL-33 group were significantly restored in the presence of miR-34a-5p antagomir (Figures 6E–H).

**Figure 7:**
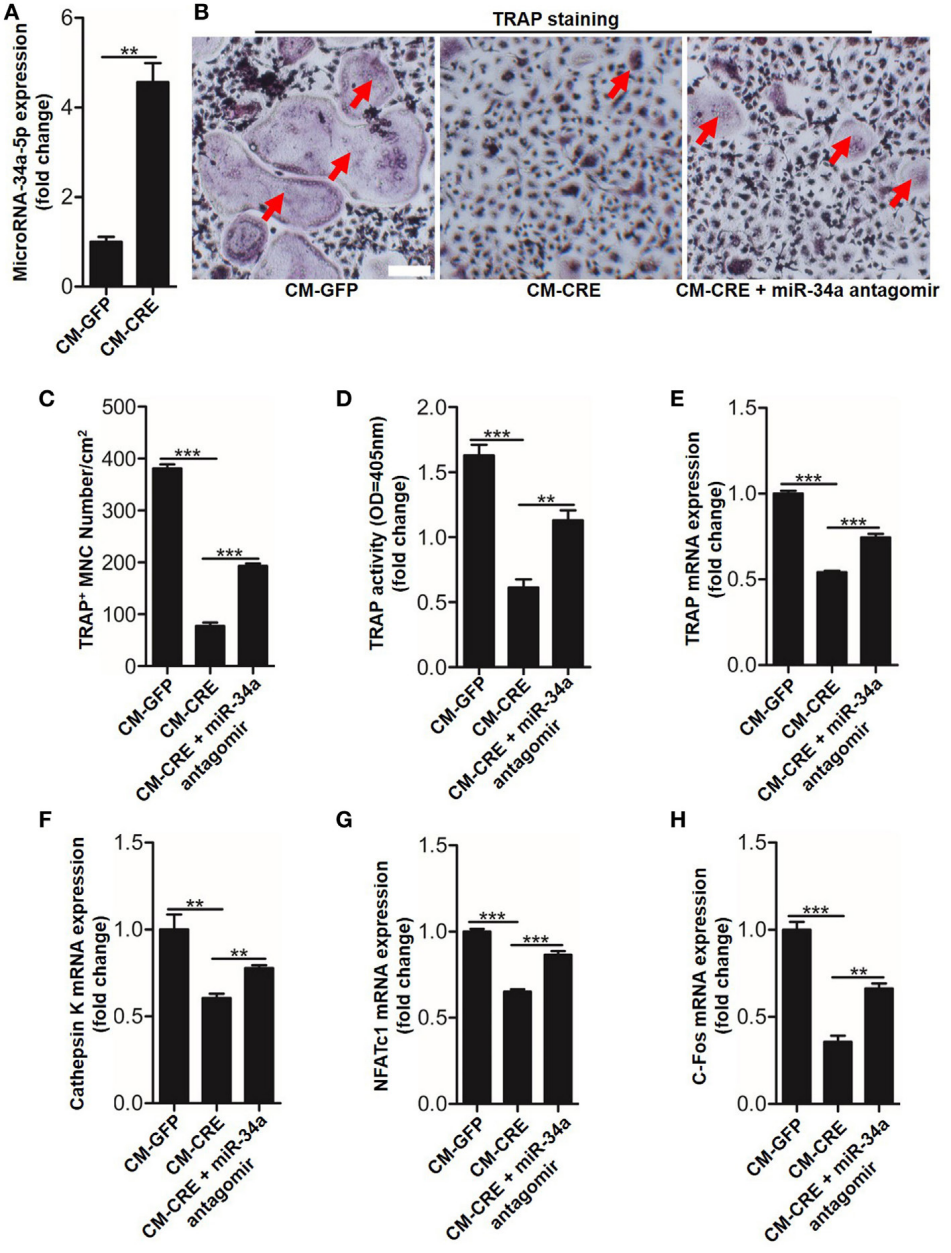
CM-CRE restrains bone marrow-derived monocytes (BMMs) osteoclastogenesis by increasing miR-34a-5p expression. **(A)** Quantification of miR-34a-5p expression induced by CM-CRE in osteoclast progenitors. **(B–D)** BMMs osteoclastic formation and tartrate-resistant acid phosphatase (TRAP) activity assay incubated with CM-CRE in the presence of miR-34a-5p antagomir. Red arrows indicated multinucleated osteoclasts. Scale bars represent 100 µm. **(E–H)** Quantification of TRAP, Cathepsin K, Nfatc1, and C-fos mRNA levels in osteoclast progenitors incubated with CM-CRE in the presence of miR-34a-5p antagomir. Scale bars represent 100 µm. **P < 0.01, ***P < 0.001. *P* values were analyzed by *t*-test in **(A)**, one-way ANOVA.

We next sought to test whether miR-34a-5p was also involved in CM-CRE-restrained osteoclast formation. Real-time RT-PCR result revealed that miR-34a-5p was significantly upregulated upon CM-CRE incubation (Figure 7A). Furthermore, TRAP staining, TRAP activity assay and the quantitation of *Trap, capthekin-K, Nfatc1*, and *C-fos* expression results demonstrated that miR-34a-5p was also crucial for CM-CRE-inhibited osteoclastogenesis (Figures 7B–H).

**Figure 8:**
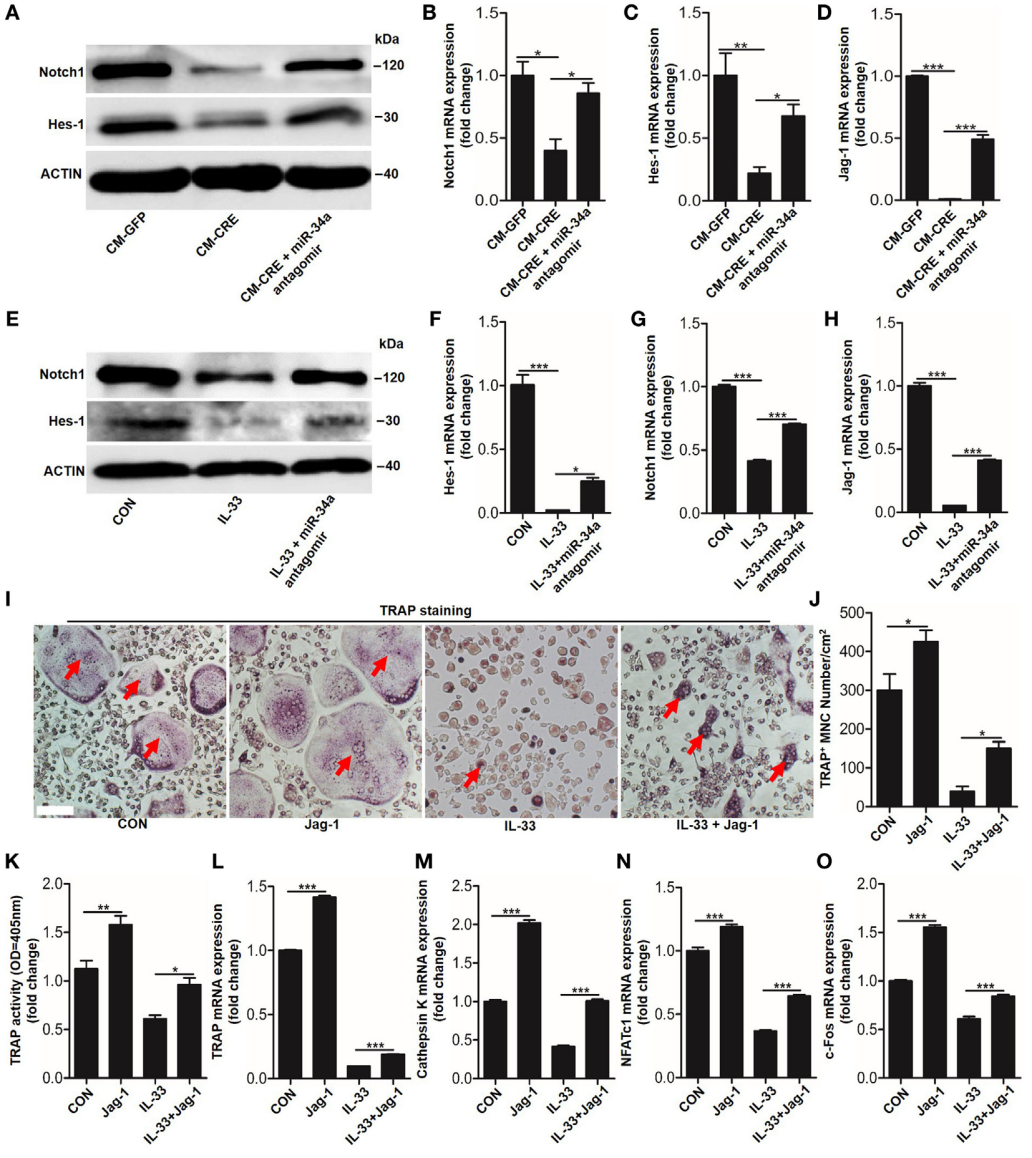
Interleukin-33 (IL-33) decreases bone marrow-derived monocytes (BMMs) osteoclastogenesis by inhibiting Notch1 activity. **(A)** Western blot analysis of Notch1 and Hes-1 expression in osteoclast progenitors incubated with CM-CRE in the presence of miR-34a-5p antagomir. **(B–D)** Quantification of Notch1, Jag1, and Hes-1 expression in osteoclast progenitors incubated with CM-CRE in the presence of miR-34a-5p antagomir. **(E)** Western blot analysis of Notch1 and Hes-1 expression in osteoclast progenitors induced by IL-33 in the presence of miR-34a-5p antagomir. **(F–H)** Quantification of Notch1, Jag1, and Hes-1 expression in osteoclast progenitors treated with IL-33 in the presence of miR-34a-5p antagomir. **(I–K)** BMMs osteoclastic formation and tartrate-resistant acid phosphatase (TRAP) activity assay after treatment with 20 ng/ml IL-33 in the presence of Jag-1. Red arrows indicated multinucleated osteoclasts. Scale bars represent 100 µm. **(L–O)** Quantification of *Trap, Cathepsin K, Nfatc1*, and *C-fos* expression in osteoclast progenitors treated with 20 ng/ml IL-33 in the presence of Jag-1. *P < 0.05, **P < 0.01, ***P < 0.001. *P* values were analyzed by one-way ANOVA.

Having observed the critical role of miR-34a-5p in CM-CRE and IL-33-inhibited osteoclastogenesis, we next sought to detect the underlying mechanism by which miR-34a-5p mediates osteoclast formation. Since several studies have described the role of Notch1 in osteoclast formation (23) and our previous work has demonstrated that miR-34a-5p mediates bone marrow stromal cell osteogenic differentiation *via* Notch1 (21), we hypothesized that miR-34a-5p regulates osteoclast formation through Notch1. Our results showed that the expression of Notch1, the Notch1 ligand Jagged1 (*Jag1*), and its target gene hairy and enhancer of split-1 (*Hes1*) in BMMs were strongly reduced by CM-CRE and IL-33. However, this effect was significantly weakened by application of miR-34a-5p antagomir (Figures 8A–H; Figure S5 in Supplementary Material). Together, these results demonstrate that miR-34a-5p is the mediator of CM-CRE and IL-33-decreased Notch1 pathway activity.

Next, we explored whether reduced Notch1 activity was essential for IL-33 inhibited-osteoclast formation. TRAP staining and TRAP activity assay results showed that activation of Notch1 by JAG1 restored IL-33-inhibited osteoclast formation (Figures 8I–K). In addition, the quantification of mRNA levels of TRAP, cathepsin-K, Nfatc1, and C-fos further demonstrated that IL-33-inhibited osteoclastogenesis by decreasing Notch1 activity (Figures 8L–O).

## Discussion

Osteoblasts have been reported to play a pivotal role in osteoclasts differentiation and function by several different mechanisms (3). Although some studies have revealed that osteoblasts HIF-1α pathway activation results in impaired osteoclastogenesis *via* the direct regulation of OPG (12, 13), whether there are other mediators are involved in osteoblast HIF-1α pathway activation-restrained osteoclasts formation still need for further investigation. Here, we proved that in addition to upregulation of OPG, HIF-1α activation in osteoblasts by *Vhl* silencing leads to increased IL-33 expression, and IL-33 plays a role in inhibiting osteoclastogenesis. Mechanistically, HIF-1α facilitates IL-33 expression by binding at −1,504/−1,500 bp on the *Il-33* promoter. IL-33 then acts through the miR-34a-5p-Notch1 pathway on BMMs to reduce their differentiation in osteoclasts. The identification of IL-33 as an intermediator of the inhibitory effect of the osteoblast HIF-1α pathway on osteoclastogenesis and the discovery of its induction and action pathways, provides a previous unknown mechanism for understanding HIF-1α pathway in bone remodeling.

As oxygen-sensitive cells, osteoblasts are exposed to mild hypoxic conditions during bone development. Therefore, it is meaningful to investigate how osteoblasts regulate the maturation of osteoclasts under hypoxia. HIFs are the main regulators of adaptive response to changes in oxygen tension (pO_2_) (24). Besides promoting osteogenesis by upregulation of VEGF, transforming growth factor β1 (TGF-β1) and insulin-like growth factor II, osteoblasts are also reported to enhance bone resorption by increasing osteoclast activity in response to hypoxia (25). Our previous study showed that activation of HIF-1α in mature osteoblasts through *Vhl*-CKO profoundly increases angiogenesis and osteogenesis, whereas mice with *Hif-1*α CKO in osteoblast had decreased bone volume and vascularity (10). Except of directly promoting bone development by increasing angiogenesis or osteoblastic activity (11), our results also suggested that activating the hypoxia/HIF-1α pathway might disturb osteoblast-osteoclast coupling to dampen osteoclast differentiation by OPG (12). Whether HIF-1α pathway activation triggers the production of other factors that are involved in vascular regeneration as well as osteoclast differentiation is still unknown. Here, we found that HIF-1 pathway activation in osteoblasts could also mediate osteoclastogenesis *via* IL-33.

Interleukin-33, also named IL-1F11, is reported to be expressed by endothelial and epithelial cells after stress and is released from cells to initiate inflammatory responses (26). IL-33 induces signal transduction by binding to a heterodimeric receptor complex consisting of interleukin-1 receptor-like 1 (ST2) and IL-1 receptor accessory protein. ST2 is mainly expressed on mast cells, Th2 cells, keratinocytes, and macrophages (27–30). In addition to functioning as a proinflammatory cytokine in the pathogenesis of asthma, atopic dermatitis, and allergic shock, the diseases characterized by Th2 inflammatory responses (26, 27, 31, 32), IL-33 was also found to be essential for the initiation of Rheumatoid arthritis, which is an autoimmune disease characterized by Th1/Th17 responses (33, 34). Recently, it was reported that IL-33 was upregulated during osteoblast differentiation and worked as a suppressor of osteoclast formation (7, 8). However, whether IL-33 is involved in osteoblast HIF-1α pathway mediated osteoclast differentiation is less known. Here, we found IL-33 is increased in *Vhl*-silenced osteoblasts *in vivo* and *in vitro*, and osteoblast HIF-1α pathway activation inhibited osteoclastogenesis partly through IL-33, as an IL-33 blocking antibody strongly reduced the inhibitory effect of CM-CRE on osteoclastogenesis in BMMs, including osteoclast formation, TRAP activity, and bone resorption activity. Mechanistically, *Vhl* deficiency leads to HIF-1α accumulation and upregulated IL-33 expression by binding to the −1,504/−1,500 bp site on the promoter of *Il-33*.

As a emerging group of small (~20 nucleotides), non-coding, single-stranded RNA molecules, miRNAs are reported to negatively regulate their target genes by inducing mRNA degradation or through inhibition of translation (35). Recently, miRNAs have emerged as important regulatory elements in bone metabolism. Besides our previous report that miR-34a-5p was involved in dexamethasone-damped BMSCs proliferation and osteogenic differentiation (21), miR-148a was reported to regulate osteoclast formation by targeting V-maf musculoaponeurotic fibrosarcoma oncogene homolog B (36). miR-503 regulates osteoclastogenesis by targeting RANK (22). However, whether miRNAs take part in IL-33-mediated osteoclastogenesis was unknown. Here, we found that IL-33 upregulated miR-34a-5p expression in a dose-dependent manner, and this increase in miR-34a-5p was essential for IL-33 and CM-CRE inhibited osteoclastogenesis as miR-34a-5p antagomir drastically reduced the inhibitory effect of IL-33 and CM-CRE on osteoclast differentiation, TRAP activity and osteoclastic bone resorption activity. Simultaneously, our data also revealed that miR-34a-5p antagomir did not completely reverse the inhibitory effects of IL-33 on osteoclast formation. Besides miR-34a-5p, we found miR-125a-5p and miR-23a-3p were also significantly upregulated in response to IL-33 stimulation, thereby, whether miR-125a-5p, miR-23a-3p and/or other miRNAs were involved in IL-33 restrained-osteoclastogenesis still need for further investigation.

It has also been reported that RANKL stimulation triggers the activation of Notch1, which is essential for bone marrow precursors to differentiate into osteoclasts (23). In the present study, we found that Notch1 accumulation induced by RANKL was significantly reduced by IL-33, and activation of Notch1 by JAG1 significantly inhibited IL-33-increased miR-34a-5p expression, as well as abundantly restored the osteoclast formation inhibited by IL-33. Therefore, our study identified a previously unknown mechanism: IL-33-miR-34a-5p pathway inhibited osteoclastogenesis *via* Notch1. However, as the results in Figure 8 revealed that Jag-1 stimulation did not completely rescue the dampened osteoclast formation induced by IL-33, which predicts that IL-33-miR-34a-5p inhibition of osteoclastogenesis could also act through an alternative pathway.

However, we would like to point out some potential limitations of our study. First, although our results revealed that IL-33 mediated osteoclastogenesis *via* the miR-34a-5p-Notch1 pathway, this conclusion is based on observations of mouse BMMs. Whether the same mechanisms are shared in human BMMs is unknown. Furthermore, the exact functional mechanisms of IL-33 on osteoclastogenesis *in vivo* require further investigation. Finally, the hypothesis that osteoblast HIF-1α pathway regulates osteoclastogenesis *via* IL-33-miR-34a-5p-Notch1 still needs to be confirmed in animal models of osteoporosis, and the clinical relevance of this pathway in osteoporosis or osteopetrosis remains to be elucidated.

In conclusion, our study uncovers that IL-33 is an intermediate factor in the cross-talk between osteoblasts HIF-1α pathway activation and osteoclasts formation. The identification of the IL-33-miR-34a-5p-Notch1 pathway provides new molecular mechanisms, which contribute to anti-osteoclastogenesis and may ultimately lead to the development of novel treatments for bone metabolism diseases.

## Ethics Statement

All animal experiments were performed in accordance with the protocol approved by the Shanghai Jiao Tong University (SJTU) Animal Care and Use Committee and in accordance with the Ministry of Science and Technology of the People’s Republic of China Animal Care guidelines.

## Author Contributions

Conceived and designed the experiments: CL, LD, and HK; performed the experiments: HK, KY, JQ, LG, YY, CG, and FW. Analyzed the data: CL and HK; contributed reagents/materials/analysis tools: LD, CL, LX, and BR. Wrote the paper: CL.

## Conflict of Interest Statement

The authors declare that the research was conducted in the absence of any commercial or financial relationships that could be construed as a potential conflict of interest.

## Abbreviations

HIF-1α, hypoxia inducible factor-1α; IL-33, interleukin-33; OPG, osteoprotegerin; BMMs, bone marrow-derived monocytes; RANKL, receptor activator of nuclear factor kappa-B ligand; VHL, von Hippel–Lindau tumor suppressor protein; PHDs, prolyl hydroxylase-domain proteins; VEGF, vascular endothelial growth factor; Ad-CRE, adenovirus expressing Cre recombinase; Ad-GFP, adenovirus expressing green fluoresence protein; TRAP, tartrate-resistant acid phosphatase; CM-GFP, culture medium of osteoblasts infected with Ad-GFP; CM-CRE, culture medium of osteoblasts infected with Ad-CRE; *Vhl*-CKO, *Vhl*-conditional knockout mice, Cre-recombinase positive mice; *Vhl*-CON, control mice, Cre-recombinase negative mice; DFO, Desferrioxamine; *Jag1*, Jagged1; *Hes1*, hairy and enhancer of split-1; N.Oc/BS, osteoclast numbers per millimeter of trabecular bone surface.
