# Supplementary material for: Osteoblast Hypoxia-Inducible Factor-1α Pathway Activation Restrains Osteoclastogenesis via the Interleukin-33-MicroRNA-34a-Notch1 Pathway
Source: Front Immunol. 2017 Oct 16;8:1312. doi: 10.3389/fimmu.2017.01312 (PMC5650688; doi:10.3389/fimmu.2017.01312)
Supplement: Supplementary file 1 [file Data_Sheet_1.PDF]

Osteoblast HIF-1α pathway activation restrains osteoclastogenesis via the IL-33-microRNA-34a-Notch1 pathway

Hui Kang[1,2#], Kai Yang[1#], Lianbo Xiao[3#], Lei Guo[1], Changjun Guo[1], Yufei Yan[1], Jin Qi[1], Fei Wang[1], Kaizhe Chen[1], Bernhard Ryffel[4], Changwei Li[1]*， Lianfu Deng[1]*

[1] Shanghai Key Laboratory for Prevention and Treatment of Bone and Joint Diseases with Integrated Chinese-Western Medicine, Shanghai Institute of Traumatology and Orthopedics, Ruijin Hospital, Shanghai Jiaotong University School of Medicine, 197 Ruijin 2nd Road, Shanghai, 200025, China.

[2] Department of Orthopedic Surgery, Shanghai Tenth People's Hospital Affiliated to Tongji University, Shanghai 200072, China.

[3] Guanghua Integrative Medicine Hospital & Institute of Arthritis Research, Shanghai Academy of Chinese Medical Sciences, Shanghai 200052, China

[4] Experimental and Molecular Immunology and Neurogenetics (INEM), UMR 7355 CNRS and University of Orleans, F-45071 Orleans-Cedex2, France.

[#] These authors contributed equally to this work

*** Corresponding author:**

Changwei Li

Shanghai Key Laboratory for the Prevention and Treatment of Bone and Joint Diseases with Integrated Chinese-Western Medicine, Shanghai Institute of Traumatology and Orthopedics, Rui Jin Hospital, Shanghai Jiao Tong University School of Medicine. Address: No. 197, Ruijin 2nd Road, Shanghai 200025, China; Zip Code: 200025; Fax: +86 21 64335742； Tel.: +86 21 64313534; E-mail: changwei393331@163.com

Lianfu Deng

Shanghai Key Laboratory for the Prevention and Treatment of Bone and Joint Diseases with Integrated Chinese-Western Medicine, Shanghai Institute of Traumatology and Orthopedics, Rui Jin Hospital, Shanghai Jiao Tong University School of Medicine. Address: No. 197, Ruijin 2nd Road, Shanghai 200025, China; Zip Code: 200025; Fax: +86 21 64335742； Tel.: +86 21 64313534; E-mail: lf_deng@126.com

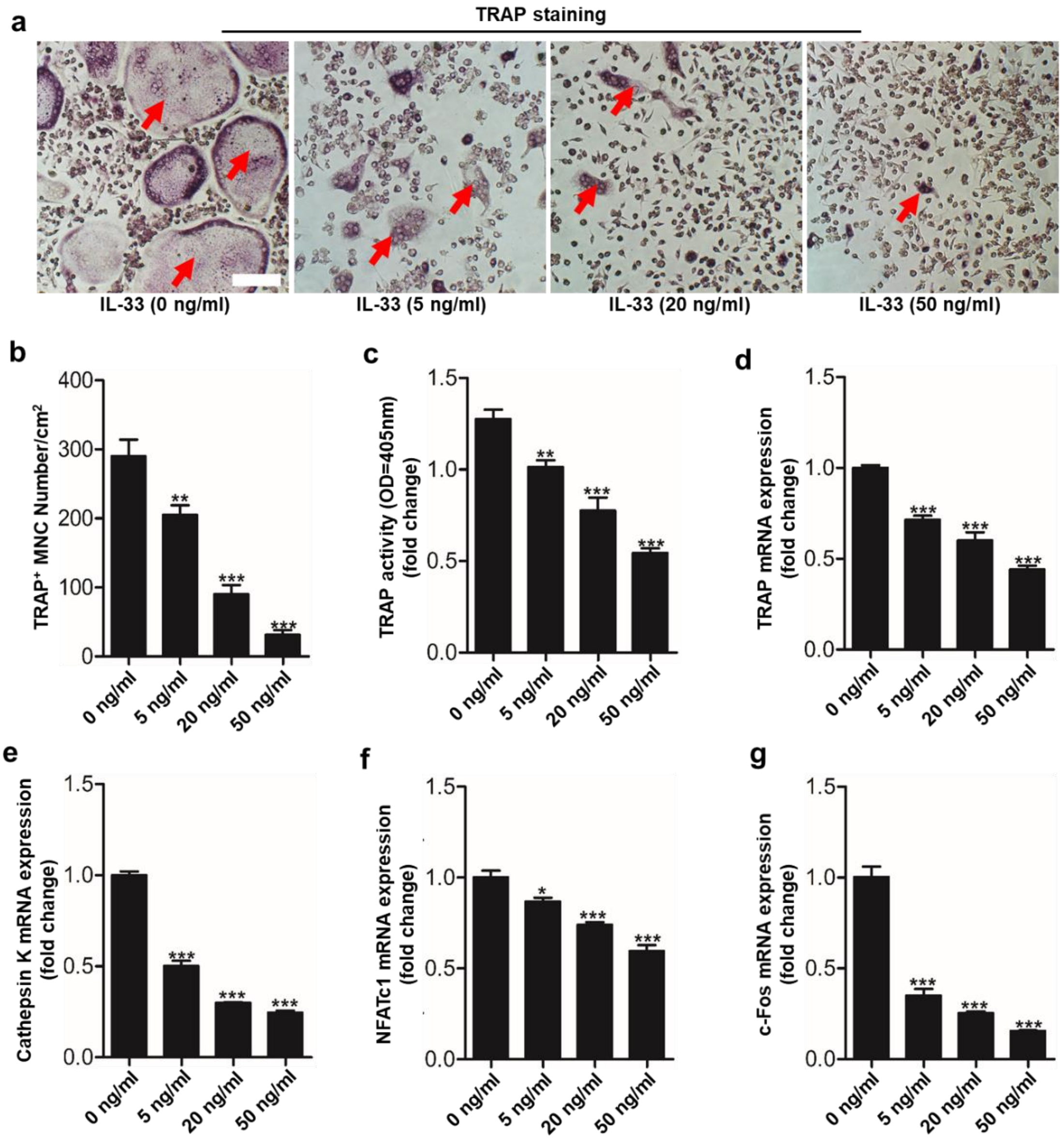

Supplementary figure 1. IL-33 inhibits bone marrow monocyte osteoclastogenesis in a dose-dependent manner. (a-c) Bone marrow monocyte osteoclastic formation and TRAP activity assay after treatment with different doses of IL-33. Red arrows indicated multinucleated osteoclasts. (d-g) Quantification of *Trap*, *Cathepsin K*, *C-fos* and *Nfatc1* mRNA levels in bone marrow monocytes treated with different doses of IL-33. Scale bars represent 100 μm. **P*<0.05, ***P*<0.01, ****P*<0.001. *P* values were analyzed by one-way ANOVA.

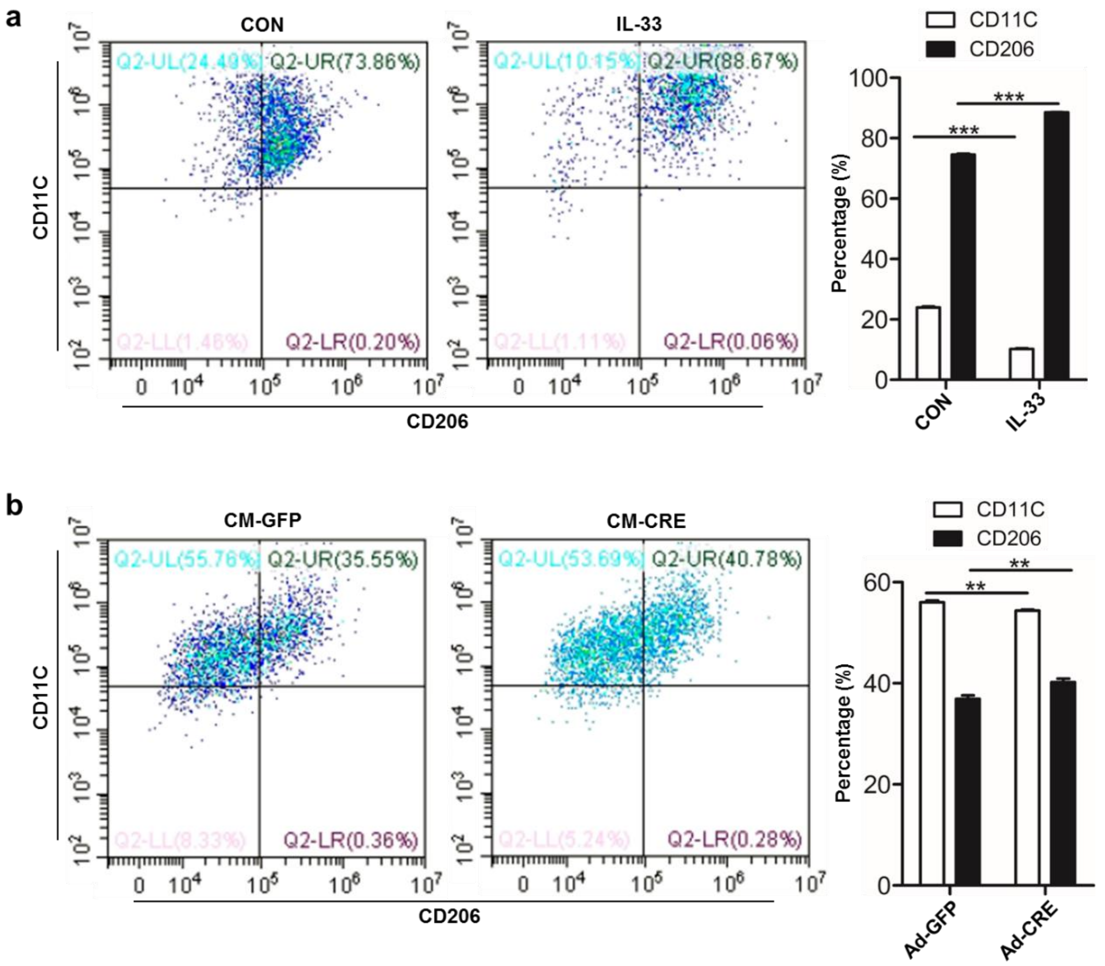

Supplementary figure 2. Bone marrow monocytes polorization indcued by IL-33, CM-GFP and CM-CRE. (a) M1 and M2 macrophage polorization from bone marrow monocyte induced by 20 ng/ml IL-33 for 72 hours. (b) M1 and M2 macrophage polorization from bone marrow monocyte induced by CM-GFP and CM-CRE for 72 hours. CM-CRE represents the culture medium of osteoblasts transfected with Ad-CRE while CM-GFP represents the culture medium of osteoblasts transfected with Ad-GFP. ***P*<0.01, ****P*<0.001. *P* values were analyzed by two-sided Student's *t*-test.

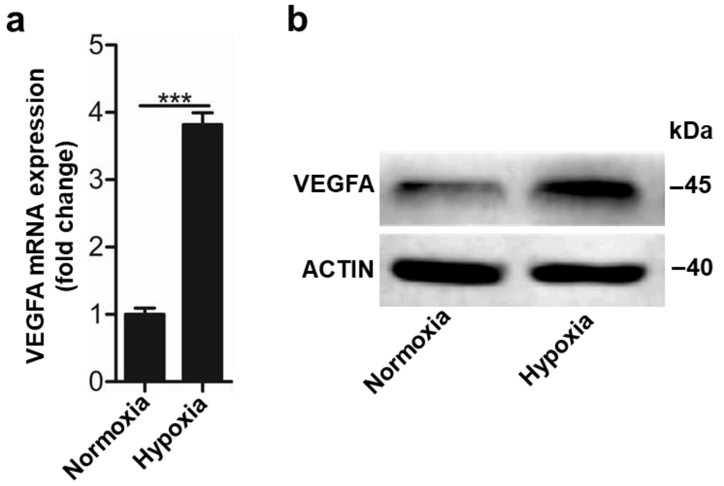

Supplementary figure 3. Hypoxia increases VEGFA expression in osteoblasts. (a) Quantification of VEGFA mRNA expression in osteoblasts induced by 1% O_2 for 24 hours. (b) Western blot analysis of VEGFA in osteoblasts induced by 1% O_2 for 24 hours. $^{***}P<0.001$. *P* values were analyzed by two-sided Student's *t*-test.

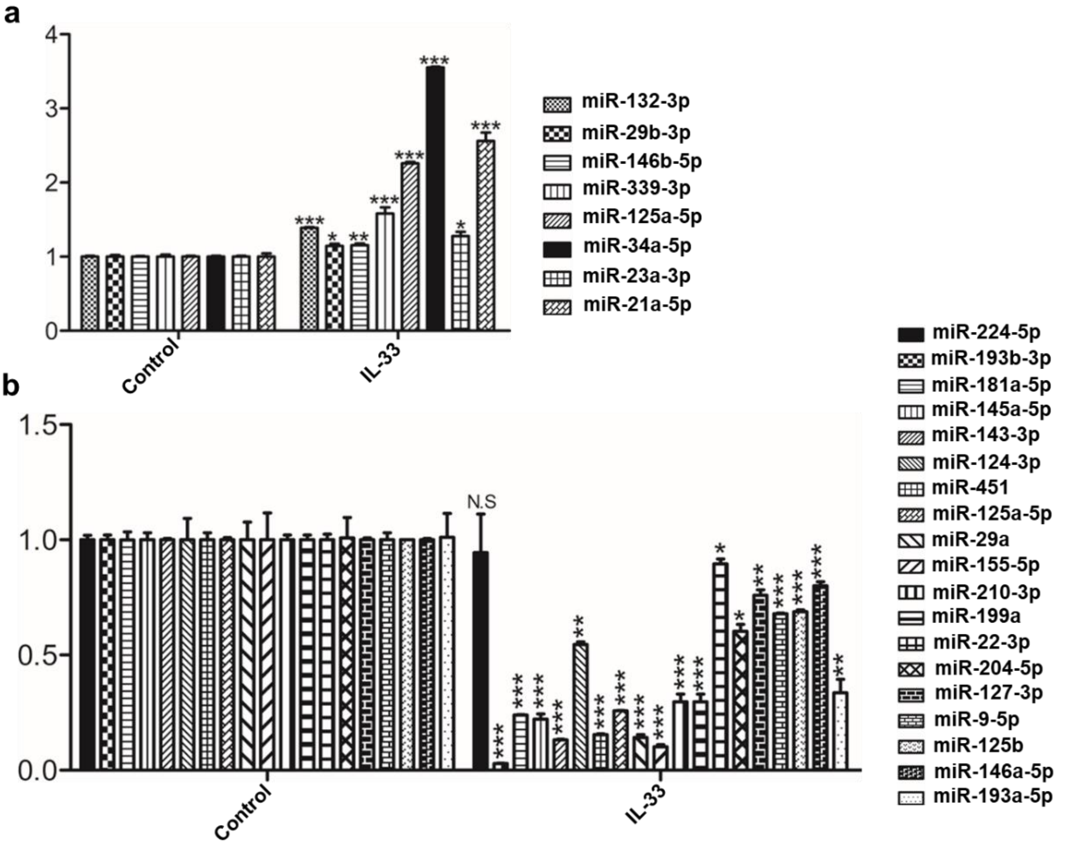

Supplementary figure 4. MicroRNAs expression induced by IL-33 in osteoclast progenitors. (a) The upregulated microRNAs detected by real time RT-PCR in osteoclast progenitors induced by 20 ng/ml IL-33. (b) MicroRNA-224-5p and the other downregulated microRNAs detected by real time RT-PCR in osteoclast progenitors induced by 20 ng/ml IL-33. *P<0.05, **P<0.01, ***P<0.001. P values were analyzed by two-sided Student's t-test.

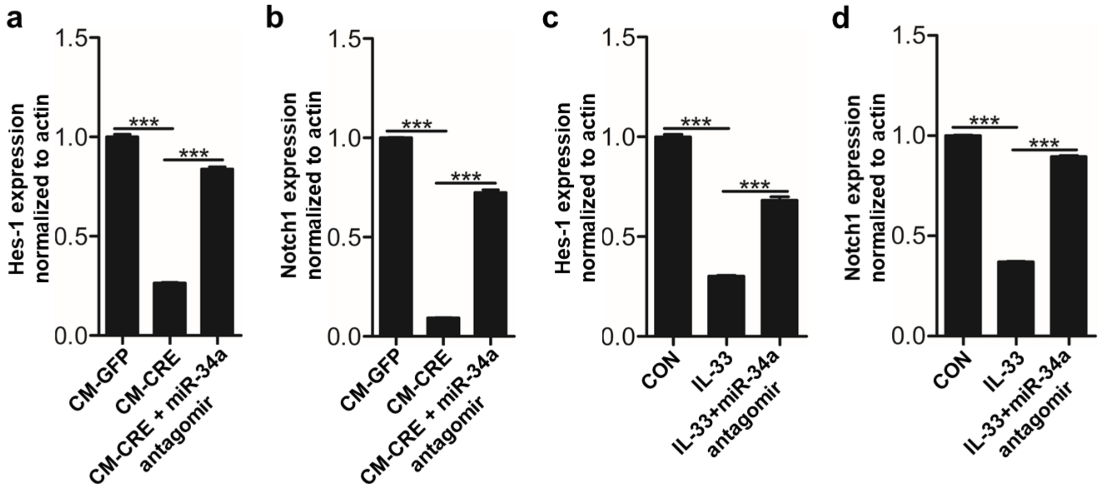

Supplementary figure 5. Hes-1 and Notch1 expression in figure 8a and 8e by normalized to GAPDH. (a & b) Hes-1 and Notch1 expression in figure 8a by normalized to actin. (c & d) Hes-1 and Notch1 expression in figure 8e by normalized to actin. ***P<0.001. P values were analyzed by one-way ANOVA.

Table 1: Primer sequences for real time RT-PCR

mmu-U6	RT: CGCTTCACGAATTTGCGTGTCAT
	F: CAAAGTGCTTACAGTGCAGGTAG
	R: CTACCTGCACTGTAAGCACTTTG
mmu-miR-132-3p	RT: GTCGTATCCAGTGCGTGTCGTGGAGTCGGCAATTGCACTGGATACGACCGACCAT
	F: GGGTAACAGTCTACAGCCATGG
	R: CAGTGCGTGTCGTGGAGT
mmu-miR-193b-3p	RT: GTCGTATCCAGTGCGTGTCGTGGAGTCGGCAATTGCACTGGATACGACAGCGGGA
	F: GGGAACTGGCCCACAAAGTCCC
	R: CAGTGCGTGTCGTGGAGT
mmu-miR-181a-5p	RT: GTCGTATCCAGTGCGTGTCGTGGAGTCGGCAATTGCACTGGATACGACACTCACC
	F: GGGAACATTCAACGCTGTCGGTG
	R: CAGTGCGTGTCGTGGAGT
mmu-miR-145a-5p	RT: GTCGTATCCAGTGCGTGTCGTGGAGTCGGCAATTGCACTGGATACGACAGGGATT
	F: GGGGTCCAGTTTTCCCAGGAATC
	R: CAGTGCGTGTCGTGGAGT
mmu-miR-29b-3p	RT: GTCGTATCCAGTGCGTGTCGTGGAGTCGGCAATTGCACTGGATACGACAACACTG
	F: GGGTAGCACCATTTGAAATCAGT

	R: CAGTGCGTGTCGTGGAGT
mmu-miR-22-3p	RT: GTCGTATCCAGTGCGTGTCGTGGAGTCGGCAATTGCACTGGATACGACAACACTG
	F: GGGAAGCTGCCAGTTGAAGAAC
	R: CAGTGCGTGTCGTGGAGT
mmu-miR-146b-5p	RT: GTCGTATCCAGTGCGTGTCGTGGAGTCGGCAATTGCACTGGATACGACAGCCTAT
	F: GGGTGAGAACTGAATTCCATAG
	R: CAGTGCGTGTCGTGGAGT
mmu-miR-339-3p	RT: GTCGTATCCAGTGCGTGTCGTGGAGTCGGCAATTGCACTGGATACGACCGGCTCT
	F: GGGTGAGCGCCTCGGCGACAGA
	R: CAGTGCGTGTCGTGGAGT
mmu-miR-204-5p	RT: GTCGTATCCAGTGCGTGTCGTGGAGTCGGCAATTGCACTGGATACGACAGGCATA
	F: GGGTTCCCTTTGTCATCCTATG
	R: CAGTGCGTGTCGTGGAGT
mmu-miR-125a-5p	RT: GTCGTATCCAGTGCGTGTCGTGGAGTCGGCAATTGCACTGGATACGACTCACAGG
	F: GGGTCCCTGAGACCCTTTAACCTG
	R: CAGTGCGTGTCGTGGAGT
mmu-miR-143-3p	RT: GTCGTATCCAGTGCGTGTCGTGGAGTCGGCAATTGCACTGGATACGACGAGCTAC
	F: GGGTGAGATGAAGCACTGTAG
	R: CAGTGCGTGTCGTGGAGT
mmu-miR-146a-5p	RT: GTCGTATCCAGTGCGTGTCGTGGAGTCGGCAATTGCACTGGATACGACAACCCAT
	F: GGGTGAGAACTGAATTCCATGG
	R: CAGTGCGTGTCGTGGAGT
mmu-miR-127-3p	RT: GTCGTATCCAGTGCGTGTCGTGGAGTCGGCAATTGCACTGGATACGACAGCCAAG
	F: GGGTCGGATCCGTCTGAGCTT
	R: CAGTGCGTGTCGTGGAGT
mmu-miR-9-5p	RT: GTCGTATCCAGTGCGTGTCGTGGAGTCGGCAATTGCACTGGATACGACTCATACA
	F: GGGTCTTTGGTTATCTAGCTGTA
	R: CAGTGCGTGTCGTGGAGT
mmu-miR-124-3p	RT: GTCGTATCCAGTGCGTGTCGTGGAGTCGGCAATTGCACTGGATACGACGGCATTC
	F: GGGTAAGGCACGCGGTGAAT
	R: CAGTGCGTGTCGTGGAGT
mmu-miR-193a-5p	RT: GTCGTATCCAGTGCGTGTCGTGGAGTCGGCAATTGCACTGGATACGACTCATCTT
	F: GGGTGGGTCTTTGCGGGCAAGA
	R: CAGTGCGTGTCGTGGAGT
mmu-miR-451	RT: GTCGTATCCAGTGCGTGTCGTGGAGTCGGCAATTGCACTGGATACGACAGGCATA
	F: GGGAAACCGTTACCATTACTGA
	R: CAGTGCGTGTCGTGGAGT
mmu-miR-503	RT: GTCGTATCCAGTGCGTGTCGTGGAGTCGGCAATTGCACTGGATACGACCTGCAGT
	F: GGGTAGCAGCGGGAACAGTAC
	R: CAGTGCGTGTCGTGGAGT
mmu-miR-29a	RT: GTCGTATCCAGTGCGTGTCGTGGAGTCGGCAATTGCACTGGATACGACTAACCGA
	F: GGGTAGCACCATCTGAAATCGG
	R: CAGTGCGTGTCGTGGAGT

mmu-miR-23a-3p	RT: GTCGTATCCAGTGCGTGTCGTGGAGTCGGCAATTGCACTGGATACGACGGAAATC
	F: GGGATCACATTGCCAGGGAT
	R: CAGTGCGTGTCGTGGAGT
mmu-miR-155-5p	RT: GTCGTATCCAGTGCGTGTCGTGGAGTCGGCAATTGCACTGGATACGACACCCCTA
	F: GGGTTAATGCTAATTGTGAT
	R: CAGTGCGTGTCGTGGAGT
mmu-miR-21a-5p	RT: GTCGTATCCAGTGCGTGTCGTGGAGTCGGCAATTGCACTGGATACGACTCAACAT
	F: GGGTAGCTTATCAGACTGATG
	R: CAGTGCGTGTCGTGGAGT
mmu-miR-125b	RT: GTCGTATCCAGTGCGTGTCGTGGAGTCGGCAATTGCACTGGATACGACTCACAAG
	F: GGGTCCCTGAGACCCTAACT
	R: CAGTGCGTGTCGTGGAGT
mmu-miR-34a-5p	RT: GTCGTATCCAGTGCGTGTCGTGGAGTCGGCAATTGCACTGGATACGACACAACCA
	F: GGGTGGCAGTGTCTTAGC
	R: CAGTGCGTGTCGTGGAGT
mmu-miR-224-5p	RT: GTCGTATCCAGTGCGTGTCGTGGAGTCGGCAATTGCACTGGATACGACAACGGAA
	F: GGGTAAGTCACTAGTGGTTCC
	R: CAGTGCGTGTCGTGGAGT
mmu-miR-210-3p	RT: GTCGTATCCAGTGCGTGTCGTGGAGTCGGCAATTGCACTGGATACGACTCAGCCG
	F: GGGCTGTGCGTGTGACAGC
	R: CAGTGCGTGTCGTGGAGT
mmu-miR-199a	RT: GTCGTATCCAGTGCGTGTCGTGGAGTCGGCAATTGCACTGGATACGACTAACCAA
	F: GGGACAGTAGTCTGCACATTGG
	R: CAGTGCGTGTCGTGGAGT
β -actin	F: GGCTGTATTCCCCTCCATCG
	R: CCAGTTGGTAACAATGCCATGT
Vhl	F: GAGGGACCCGTTCCAATAATG
	R: GTGCCCGGTGGTAAGATCG
TRAP	F: CACTCCCACCCTGAGATTTGT
	R: CATCGTCTGCACGGTTCTG
c-Fos	F: CGGGTTTCAACGCCGACTA
	R: TTGGCACTAGAGACGGACAGA
Cathepsin K	F: GAAGAAGACTCACCAGAAGCAG
	R: TCCAGGTTATGGGCAGAGATT
DC-STAMP	F: GGGGACTTATGTGTTTCCACG
	R: ACAAAGCAACAGACTCCCAAAT
NFATc1	F: GACCCGGAGTTCGACTTCG
	R: TGACACTAGGGGACACATAACTG
V-ATPase d2	F: CAGAGCTGTACTTCAATGTGGAC
	R: AGGTCTCACACTGCACTAGGT
Mmp9	F: CTGGACAGCCAGACACTAAAG
	R: CTCGCGGCAAGTCTTCAGAG
Il-33	F: TCCAACTCCAAGATTTCCCCG

	R: CATGCAGTAGACATGGCAGAA
Notch1	F: GATGGCCTCAATGGGTACAAG
	R: TCGTTGTTGTTGATGTCACAGT
Jag-1	F: CCTCGGGTCAGTTTGAGCTG
	R: CCTTGAGGCACACTTTGAAGTA
Hes-1	F: CCAGCCAGTGTCAACACGA
	R: AATGCCGGGAGCTATCTTTCT
